# Compounded Nonsterile Preparations and FDA-Approved Commercially Available Liquid Products for Children: A North American Update

**DOI:** 10.3390/pharmaceutics14051032

**Published:** 2022-05-10

**Authors:** Richard H. Parrish, Lisa D. Ashworth, Raimar Löbenberg, Sandra Benavides, Jeffrey J. Cies, Robert B. MacArthur

**Affiliations:** 1 Department of Biomedical Sciences, Mercer University School of Medicine, Columbus, GA 31902, USA; 2 Department of Pharmacy Services, Children’s Health System of Texas, Dallas, TX 75235, USA; rlisadashworth@gmail.com; 3 Faculty of Pharmacy and Pharmaceutical Sciences, University of Alberta, Edmonton, AB T6G 2R3, Canada; raimar@ualberta.ca; 4 School of Pharmacy, Philadelphia College of Osteopathic Medicine, Suwanee, GA 30024, USA; sandra.b.caballero@gmail.com; 5 Department of Pediatrics, Drexel University College of Medicine, Philadelphia, PA 19129, USA; jeffrey.cies@towerhealth.org; 6 Department of Pharmacy Services, St. Christopher’s Hospital for Children/Tower Health, Philadelphia, PA 19134, USA; 7 Department of Pharmacy Services, Rockefeller University Hospital, New York, NY 10065, USA; rmacarthur@researchpharmacy.com

**Keywords:** active pharmaceutical ingredient, compounded drug, compounding, extemporaneous formulation, manufactured material, medication, monograph, pediatric, reference standards

## Abstract

The purpose of this work was to evaluate the suitability of recent US Food and Drug Administration (US-FDA)-approved and marketed oral liquid, powder, or granule products for children in North America, to identify the next group of Active Pharmaceutical Ingredients (APIs) that have high potential for development as commercially available FDA-approved finished liquid dosage forms, and to propose lists of compounded nonsterile preparations (CNSPs) that should be developed as commercially available FDA-approved finished liquid dosage forms, as well as those that pharmacists should continue to compound extemporaneously. Through this identification and categorization process, the pharmaceutical industry, government, and professionals are encouraged to continue to work together to improve the likelihood that patients will receive high-quality standardized extemporaneously compounded CNSPs and US-FDA-approved products.

## 1. Introduction

Since the publication of our last paper, a number of novel, commercially scaled pediatric formulations have been approved by the FDA that incorporate candidate molecules (active pharmaceutical ingredients—API), which in the past were primarily available only as extemporaneously compounded nonsterile preparations (CNSPs) [1]. In that paper, a list of 16 candidate active pharmaceutical ingredients (APIs) were selected from the universe of CNSPs described in the professional literature, which were commonly dispensed in pediatric clinical practice. Bearing in mind the guidance from the U.S. Food and Drug Administration (US-FDA) that discourages the compounding of drug preparations that are “essentially copies of approved products”, pharmacists should refrain from compounding any preparation that is now available in an approved finished dosage form appropriate for an individual patient [2]. The following discussion illustrates the variety and formulation characteristics of seven recently marketed mass-produced final oral liquid dosage forms submitted to and approved by the US-FDA under section 505(b)(2) of the Federal Food, Drug, and Cosmetics Act [3]. Additionally, based on the author’s experience and expertise, this report will: (1) evaluate the appropriateness of these newly marketed products for use in neonatal and pediatric populations: (2) identify the next wave of candidate APIs using the algorithm previously developed and described in the manuscript; (3) propose two lists of API candidates: (a) those that are listed currently as compounding monographs in the United States Pharmacopeia (USP) Compounding Compendium (CC) and should be considered for development as approved mass produced finished liquid dosage forms [4]; and (b) those not currently listed in USP CC that have limited mass market potential. Currently listed monographs should remain in the USP CC to provide guidance globally as well as to mitigate the impacts of drug shortages on the approved pharmaceutical supply chain.

## 2. FDA-Approved Manufactured Dosage Forms Marketed in the U.S. since 2016

Several authors recently have reviewed the array of pediatric oral formulations on the worldwide market and in development, and it has been suggested that the future of formulation development for children lies in mini-tablets and other solid dispersible dosage forms [5,6,7,8]. Many, if not most, of these dose forms provide only “close enough” dosing flexibility for individual patients. However, “close enough” often is not good enough, especially for the most vulnerable of patients [9]. In our experience, how to measure exact milligram per kilogram (mg/kg) doses from these solid orals in the in-patient setting would be problematic, considering that the pharmacies in most U.S. pediatric institutions provide a measured, ready-to-give liquid dose form for the nurse to administer for improved patient safety. Table 1 outlines the eight liquid finished dosage forms from the AA list that have become available since 2016, including two amlodipine salts.

## 3. Suitability of Recently Marketed Manufactured Liquids for Children

USP defines excipients, often called ’inactive ingredients’, “(As comprising) everything except the active pharmaceutical ingredients (APIs). Excipient functions range from helping to guarantee the stability and bioavailability of the API to the drug product’s manufacturability to its texture and taste. Excipients are major components of almost all drugs, as well as foods, cosmetics, and dietary supplements” [10]. For children, not all inactive ingredients are inactive. Because a number of excipients are inappropriate for children, the European Pediatric Formulation Initiative (EPFI) and United States Pediatric Formulation Initiative (USPFI) have created a searchable common database to assist manufacturers and compounders alike in identifying age-suitable excipients. Common excipients and preservatives found in the labeling of manufactured products marketed in the US include water, citric acid, glycerin, methyl- and propylparaben, sodium benzoate, and sodium citrate, as well as various forms of cellulose, sugars and sweeteners, and sugar alcohols. Based on the EPFI’s S.T.E.P database and PubChem, as well as a recent comprehensive review, Table 2 lists recently manufactured oral liquid products, their excipients and preservatives, and comments about the suitability for use in pediatric patients [11]. Caution should be exercised in the use of these newly approved oral liquids, especially in neonates.

## 4. Additional APIs Marketed in Manufactured Oral Liquid or Granule Dosage Forms

An additional 20 APIs have been FDA-approved and marketed in the US in an oral liquid or granule or powder for reconstitution (Table 3). Of the products listed in Table 3, several are approved for adult populations only (**bolded**), and their suitability for use as off-label treatment in children needs to be established. Of note, Azurity Pharmaceuticals has INDs submitted for two antiepileptic medications in oral liquids—lamotrigine and zonisamide. Two other products, topiramate 25 mg/mL (Eprontia™) and baclofen 5 mg/mL oral liquid formulations, were recently FDA-approved. Of note, an unapproved but marketed metronidazole 50 mg/mL product also became available as a compounding kit from Azurity.

## 5. The Next Wave of Oral Liquid Formulation Development—Formulation Considerations

The following nine APIs were included on the list of 16 and represent the next wave of candidates for conversion from extemporaneously CNSPs to commercially available FDA-approved finished liquid dosage form products, in order of their potential for development as a commercial market. Those CNSPs include ursodiol, bosentan, captopril, pantoprazole, valacyclovir, clopidogrel, acetazolamide, warfarin, and nifedipine. The suggested concentrations of oral liquid dosage forms are based on four factors: (1) the usual dosage range; (2) the maximum adult dose and volume (not to exceed 20 mL); (3) the expected water solubility; and (4) the standardized concentration for the extemporaneously compounded preparation, if applicable (Table 4).

- Ursodiol or ursodeoxycholic acid (UDCA) [12,13,14,15,16,17,18,19] inhibits the hepatic synthesis and secretion of cholesterol and its intestinal absorption. It is indicated in primary biliary cirrhosis and for the prevention and treatment of gallstones. It is BCS class II due to low solubility and high permeability. Development of an oral liquid may be facilitated using methylcellulose and glycerin as excipients and via incorporation into polymeric nanoparticle carriers [20,21].
- Bosentan [22,23], a hazardous medication, is a sulfonamide-derived, dual endothelin receptor antagonist used in the treatment of pulmonary arterial hypertension [22,24]. It belongs to BCS class II, and is available in solid and quadrisected dispersible tablets. Its oral bioavailability may be increased through nanosuspension [25] and water-rich co-solvent mixtures using propylene glycol [26].
- Captopril, [4,27,28,29,30,31,32,33,34] is an angiotensin I-converting enzyme inhibitor (ACE-I) indicated in the treatment of heart failure and hypertension [34,35,36]. It is a BCS class I agent and is freely soluble in water (160 mg/mL), although its stability is limited due to disulfide formation. Fast-dispersing tablet formulations in 2.5 and 10 mg strengths for reconstitution have been suggested [37].
- Pantoprazole [4,38] is a proton pump inhibitor prodrug in the benzimidazole family with a provisional BCS class III designation due to its high solubility and low permeability. It is currently available in a delayed release granule for the preparation of an oral suspension used to treat erosive esophagitis, gastroesophageal reflux disease, and Zollinger–Ellison syndrome. Dividing the granule formulation into smaller doses in children is not recommended in the product’s labeling. Formulation options include alginate–pectin polymeric raft-forming systems and divisible buccal films [39,40,41].
- Valacyclovir [4,42,43,44,45] is a valyl ester prodrug that is converted to acyclovir, and is indicated for herpes labialis and zoster in children. A BCS class III agent, its stability in solution is pH-dependent at concentration ranges from 2.2 to 174 mg/mL [46]. Valacyclovir tablet product labeling includes instructions for preparing a CNSP at concentrations of 25 and 50 mg/mL in 100 mL quantities, using a suspension-structured vehicle and cherry flavor to mask the bitter taste. This product has beyond use dating of up to 28 days when stored under refrigeration. However, solution formulations using powdered API with combinations of glycerol and maltodextrin have been suggested [47,48].
- Clopidogrel [4,49,50,51], a prodrug activated in two steps primarily by CYP 2C19, is a BCS class II agent with a bioavailability of about 50%. Its uses in children are for arterial ischemic stroke, heart disease, and management of endovascular stents [52,53]. Stabilization with stearoyl polyoxylglycerides (Gelucire^®^ 50/13) and polyoxyethylated castor oil (Cremophor^®^ RH40) have been suggested for self-emulsifying oral drug delivery formulations to improve the bioavailability and storage duration [54].
- Acetazolamide [4] is a potent inhibitor of the enzyme carbonic anhydrase, which catalyzes the reversible hydration of carbon dioxide and dehydration of carbonic acid, resulting in the renal loss of bicarbonate anion, sodium, and water. Its primary uses in children are for the treatment of metabolic alkalosis, seizure disorder, glaucoma (topically), and intracranial hypertension [55,56,57,58]. It is available as a lyophilized powder for injection. Acetazolamide is very slightly soluble in water (BCS class IV), and its oral formulation bioavailability may be enhanced through application of mucoadhesive nanoparticles and the use of spray drying techniques [59,60,61].
- Warfarin is an epoxide reductase inhibitor of the synthesis of vitamin K-dependent clotting factors, including the anticoagulant proteins C and S, as well as factor II, VII, IX, and X. Its indications include the prophylaxis and treatment of venous thromboembolism, pulmonary embolism, and complications associated with atrial fibrillation, myocardial infarction, and ischemic stroke. Warfarin is listed as BCS class I. While the labeled stability of a reconstituted intravenous injection is 4 h, more dilute oral solutions (1 mg/mL in aqueous media) may have longer stability [62,63]. The use of a semisolid extrusion of orodispersible hydroxypropylcellulose films created through the use of 3D printers may hold promise for both the preparation and individualization of doses [63,64].
- Nifedipine [65] is a dihydropyridine calcium channel blocker formulated as a liquid-filled capsule. It is indicated for chronic hypertension and vasospastic or chronic unstable angina [34,35]. It is BCS class II with poor water solubility, and undergoes extensive first pass metabolism. The formulation of a powder for reconstitution may be facilitated by reductions in particle size through high-pressure homogenization or fabricated nanosponge encapsulation [32,66,67,68,69].

**Table 4:** Next-wave mass-manufactured API candidates.

Medication	Dosing Range in Children (mg/kg/day)	Solubility in Water at RT and Neutral pH (mg/mL)	Suggested Mass Production Concentration	Suggested Mass Production Liquid Dose Form
**ursodiol**	10–40	0.02	60 mg/mL *	Nanoparticle suspension
**bosentan**	3–4	0.43	6.25 mg/mL	Powder for reconstitution
**captopril**	0.02–0.3	160	1 mg/mL *	Powder for reconstitution
**pantoprazole**	0.5–1	0.048	2 mg/mL	Powder for reconstitution
**valacyclovir**	60–120	174	50 mg/mL *	Solution
**clopidogrel**	0.2	0.051	5 mg/mL	Powder for reconstitution
**acetazolamide**	8–30	0.9	25 mg/mL	Nanoparticle powder for reconstitution
**warfarin**	0.05–0.35	0.017	1 mg/mL	Powder for reconstitution
**nifedipine**	1–2	0.0059	4 mg/mL *	Nanoparticle powder for reconstitution

(Note: * indicates ASHP standardized concentration [70]).

### 5.1. Additional API Candidates with High Potential for Development as Commercially Available FDA-Approved Finished Liquid Dosage Forms

Table 5 lists an additional 26 mass production candidate APIs, eight of which are on the hazardous drugs list [71]. BCS class I APIs with the highest mass marketing potential include hydroxyurea, allopurinol, flecainide, and cyclophosphamide. For BCS class II, amiodarone, carvedilol, isradipine, quetiapine, and losartan have the highest potential marketability. In BCS class III, clonidine, hydralazine, apixaban, atenolol, and ganciclovir top the list. Finally, BCS class IV API include hydrochlorothiazide and azathioprine. Hazardous APIs with mass marketing potential included hydroxyurea, ganciclovir, cyclophosphamide, and azathioprine. The commercial availability of finished liquid dosage forms for hazardous medications for oral administration affords an opportunity to reduce overall inadvertent toxic exposure due to dose manipulation in institutional settings and at home, irrespective of other evaluation criteria. These APIs:

- Have the highest marketing potential for pediatric populations in terms of off-label use in prioritized therapeutic categories (antiarrhythmics, antibiotics, antihypertensives, antineoplastics, central nervous system agents, and proton pump inhibitors) [72];
- Appear on the FDA and World Health Organization list of essential medicines [73];
- Have a standardized concentration identified on the ASHP Standardize 4 safety list [70,74];
- Are available in an intravenous formulation that would support potential oral formulation feasibility; and
- Have a pediatric Biopharmaceutical Classification System (BCS) classification indicating relative ease of generating suitable oral liquid formulations intended primarily for children [75].

BCS class II and IV APIs have low solubility and high or low gastrointestinal permeability, and several techniques have been forwarded to address them. In many cases, the optimal solubility–permeability balance is based on the extent to which membrane–aqueous partitioning is maximized; that is, the intersection where both are at their highest relative values [76]. Formulation methods to increase either solubility or dissolution rates for BCS class II and IV APIs identified in the literature include:

- 3D printing [63,77,78,79];
- Amorphous solid dispersion [59,80,81];
- Complexation [82,83];
- Fusion [84,85];
- Hot-melt extrusion [84,86];
- Lipid microemlusion [54];
- Lyophilization [84,87,88];
- Micelles [87];
- Nanosizing [60,89]; and
- Spray drying [59,60].

Excipients that have been shown to increase gastrointestinal tract permeability include:

- Cyclodextrins [67,82];
- Surfactants [53,88]; and
- Cosolvents [90].

A brief product profile for the APIs with the highest potential for development as commercially available FDA-approved finished liquid dosage forms includes: (1) clinical pharmacology/PK/PD/PG; (2) indications and dosing regimens; and (3) clinical pharmaceutics and potential formulation characteristics.

#### 5.1.1. High Solubility/High Permeability (BCS Class I)

- Hydroxyurea [91] is an antimetabolite used to treat sickle cell anemia crisis; management of melanoma; resistant chronic myelocytic leukemia; and recurrent, metastatic, or inoperable carcinoma of the ovaries. It inhibits DNA synthesis through the inhibition of ribonucleoside diphosphate reductase. It is well absorbed orally and the water solubility is 100 mg/mL. An oral liquid with a concentration of 100 mg/mL has been studied [92].
- Allopurinol is a xanthine oxidase inhibitor used to reduce urinary and serum uric acid concentrations in patients with gout, recurrent calcium oxalate calculi, and various malignancies. Children 6 to 10 years of age with secondary hyperuricemia associated with malignancies may be given 300 mg allopurinol daily, while those under 6 years are generally given 150 mg daily. The response is evaluated after approximately 48 h of therapy and a dosage adjustment is made if necessary. The solubility in water at room temperature is between 0.48 and 0.57 mg/mL. A 500 mg lyophilized injection is available. Hydrophilic carriers such as polyvinylpyrrolidone/polyethylene glycol 6000 at ratios of 1:1, 1:2, and 1:4 (drug to carrier ratio) have been shown to increase aqueous solubility [80].
- Flecainide [93] is a class Ic antiarrhythmic agent used to manage atrial fibrillation and paroxysmal supraventricular tachycardias (PSVT). Its water solubility is 48.4 mg/mL at 37 °C. Dosing in children is usually less than 100 mg per dose. A 20 mg/mL formulation using bulk powder and purified water and simple syrup (50:50) resulted in a transparent solution [94].
- Cyclophosphamide [95] is an alkylating nitrogen mustard antineoplastic and immunosuppressive agent that must be activated in the liver to aldophosphamide. In addition to solid tumors, it is also indicated for the treatment of biopsy-proven minimal change nephrotic syndrome and membranous nephritis in pediatric patients [96]. For nononcologic indications, oral cyclophosphamide dosing usually in the range of 2.5 to 3 mg/kg daily for a period of 60 to 90 days is recommended for both initial and maintenance dosing, and can be used in place of a low-dose intravenous formulation. It is soluble in water at between 10 and 50 mg/mL, and liquid formulations using the lyophilized intravenous formulation may be prepared in a 10 mg/mL concentration [97].

#### 5.1.2. Low Solubility/High Permeability (BCS Class II)

- Amiodarone, considered a class III antiarrhythmic with α- and β-receptor antagonism, is a benzofuran derivative indicated for initiation of treatment and prophylaxis of frequently recurring ventricular fibrillation (VF) and hemodynamically unstable ventricular tachycardia (VT) in patients that are refractory to other therapy. Most patients will require this therapy for 48 to 96 h, but amiodarone may be safely administered for longer periods if necessary. Pediatric dosing ranges from 10 to 15 mg/kg/day in 1 to 2 divided doses/day for 4 to 14 days or until adequate control of arrhythmia or prominent adverse effects occur. Dosage should then be reduced to 5 mg/kg/day given once daily for several weeks. If arrhythmia does not recur, reduce to the lowest effective dosage possible. Usual daily minimal dose: 2.5 mg/kg/day; maintenance doses may be given for 5 or 7 days/week. Amiodarone is available in a 50 mg/mL intravenous injection, exhibits 0.72 mg/mL solubility in water, and is highly lipophilic. A 5 mg/mL formulation at pH = 4 with cherry flavoring has been suggested [33].
- Carvedilol [12] is a racemic mixture where the S(-) enantiomer is a beta adrenoceptor blocker and the R(+) enantiomer is both a beta and alpha-1 adrenoceptor blocker. It is currently used to treat heart failure, left ventricular dysfunction, and hypertension. Pediatric dosing ranges from 0.4 to 0.8 mg/kg/day in 2 divided doses. It is virtually insoluble in water, and is highly lipophilic. A 1–1.25 mg/mL oral suspension has been suggested [31].
- Isradipine [35,36] belongs to the dihydropyridine (DHP) class of calcium channel blockers (CCBs), the most widely used class of CCBs. It is structurally related to felodipine, nifedipine, and nimodipine, and is the most potent calcium channel blocking agent of the DHP class. Isradipine binds to calcium channels with high affinity and specificity and inhibits calcium flux into cardiac and arterial smooth muscle cells. It exhibits greater selectivity towards arterial smooth muscle cells owing to alternative splicing of the alpha-1 subunit of the channel and increased prevalence of inactive channels in smooth muscle cells. Isradipine may be used to treat mild to moderate essential hypertension. Pediatric dosing ranges from 0.15 to 0.2 mg/kg/day divided every 6 to 8 h with a maximum dosage of 0.8 mg/kg/day, not to exceed 20 mg/day. It is practically insoluble in water. A 1 mg/mL suspension has been compounded since its initial marketing [98].
- Quetiapine is a dopamine type 2 and serotonin 2A receptor antagonist and binds to the norepinephrine transporter. Additional effects of quetiapine, including somnolence, orthostatic hypotension, and anticholinergic effects, may result from the antagonism of histamine-1, adrenergic α1, and muscarinic-1 receptors, respectively. It is used in the management of bipolar disorder, schizophrenia, major depressive disorder, and delirium [99]. Quetiapine is rapidly and well absorbed after administration of an oral dose, and a steady state is achieved within 48 h. It is metabolized by CPY 2D6 and 3A4. The water solubility of quetiapine is 0.6 mg/mL with a pKa of 7.06. Pediatric dosing ranges from 0.5 to 6 mg/kg/day. Nanotechnology formulations of 2.5, 5, 10, 20 and 40 mg/mL have been suggested [100,101].
- Losartan [35] is an angiotensin receptor antagonist indicated to treat hypertension in patients older than 6 years, to reduce the risk of stroke in patients with hypertension and left ventricular hypertrophy, and to treat diabetic nephropathy with elevated serum creatinine and proteinuria in patients with type 2 diabetes and hypertension. It reversibly and competitively prevents angiotensin II binding to the AT1 receptor in tissues like vascular smooth muscles and the adrenal gland. The usual recommended starting dose is 0.7 mg/kg once daily (up to 50 mg total), and dosages above 1.4 mg/kg (or in excess of 100 mg) daily have not been studied in pediatric patients. Losartan has an 8 mg/mL water solubility level with a pKa of 5.5. A 5 mg/mL formulation has recently been studied [102], and directions for the preparation of a 2.5 mg/mL suspension are contained in the package insert [103].

#### 5.1.3. High Solubility/Low Permeability (BCS Class III)

- Clonidine [104,105,106,107,108] is an imidazole derivate that acts as an agonist of alpha-2 adrenoceptors used to treat hypertension and severe cancer pain, among other conditions, and to treat withdrawal symptoms from various substances. It is available in a 0.1 mg/mL liquid solution for injection and is insoluble in water. Its bioavailability is between 55 and 87%, and the primary metabolism includes hydroxylation via CYP2D6, CYP1A2, CYP3A4, CYP1A1, and CYP3A5 enzymes. The usual pediatric dose range is between 5 and 10 mcg/kg/day orally in divided doses every 8 to 12 h then titrated based on clinical response, with a maximum dose of 25 mcg/kg/day or 0.9 mg/day.
- Hydralazine [35] is a direct-acting vasodilator that is used as an antihypertensive agent. It inhibits the phosphorylation of myosin protein and chelation of trace metals required for smooth muscle contraction, resulting in increases in heart rate, stroke volume, and cardiac output. Available in a 20 mg/mL injection solution, hydralazine is freely soluble in water. The initial dose is 0.75 mg/kg/day in 4 divided doses, with gradual increases over 3 to 4 weeks to a maximum of 7.5 mg/kg/day or 200 mg/day. Taking oral hydralazine with food improves the bioavailability of the drug. A 4 mg/mL suspension using crushed hydralazine tablets has been suggested [49].
- Apixaban [109] is an oral, direct, and highly selective factor Xa (FXa) inhibitor of both free and bound FXa, as well as prothrombinase, independent of antithrombin III, for the prevention and treatment of thromboembolic diseases. Children 12 to <18 years old weighing less than 40 kg have received an apixaban dose of 0.2 mg/kg twice daily for 7 days followed by 0.1 mg/kg twice daily, whereas children at the same age weighing more than 40 kg receive the adult VTE treatment dose (i.e., 10 mg twice daily for 7 days followed by 5 mg twice daily). Apixaban is approximately 50% bioavailable, and is mainly metabolized by cytochrome CYP 3A4 and to a lesser extent by CYP1A2, CYP2C8, CYP2C9, CYP2C19, and CYP2J2. It has a water solubility of 0.11 mg/mL. A 0.25 mg/mL suspension with a 7-day stability has been reported [110].
- Atenolol [36] is a synthetic beta-1 selective blocker used in the management of hypertension and chronic angina, and to reduce mortality in known or suspected myocardial infarction in hemodynamically stable patients. It is available in a liquid injection at a concentration of 0.5 mg/mL. Approximately 50% of an oral dose is absorbed from the gastrointestinal tract, the remainder being excreted unchanged in the feces. Its water solubility is 13.3 mg/mL. The typical dosage range for children is 0.5–1 mg/kg/day given once daily or divided in 2 doses per day with a maximum dose of 2 mg/kg/day [111].
- Ganciclovir, a DNA polymerase inhibitor prodrug used to treat cytomegalovirus (CMV) and herpetic keratitis of the eye and to prevent CMV in organ transplantation, must be converted to the active form by a virus-encoded cellular enzyme, thymidine kinase [112]. Oral administration follows intravenous induction. It has a solubility of 3 mg/mL in water with pKa values of 2.2 and 9.4. Pediatric dosing in normal renal function ranges 20–40 mg/kg or 500–700 mg/m^2^ every 8 h [113,114].

#### 5.1.4. Low Solubility/Low Permeability (BCS Class IV)

- Hydrochlorothiazide [34,115] is a thiazide diuretic used alone or in combination for the management of edema associated with congestive heart failure, hepatic cirrhosis, nephrotic syndrome, acute glomerulonephritis, chronic renal failure, and hypertension. Hydrochlorothiazide acts on the proximal region of the distal convoluted tubule, inhibiting reabsorption by the sodium-chloride symporter, also known as solute carrier family 12 member 3 (SLC12A3). It has a water solubility level of 0.7 mg/mL. Because of its poor oral absorption, several novel dosage forms have been proposed, including orally disintegrating mini-tablets, liquid complexations with cyclodextrin, and nanostructured lipid carriers [116,117,118].
- Azathioprine, a prodrug of 6-mercaptopurine, is used to treat inflammatory conditions such as rheumatoid arthritis, systemic lupus erythematosus, and inflammatory bowel diseases, as well as being an immunosuppressant in the prevention of renal transplant rejection [119]. The usual daily dose ranges from 1 to 5 mg/kg orally. It is insoluble in water, with a pKa of 7.8. Both 10 mg/mL and 50 mg/mL suspensions have been studied [49,120].

### 5.2. Extemporaneous CNSP APIs Included in USP CC Monographs

Extemporaneous CNSP APIs included in USP CC monographs that could be developed as FDA-approved manufactured products, as well as those without a mass market, are outlined below [121]. The list is broken down into those APIs whose compounding recipes are found currently (1) in the USP CC and (2) those available in other sources. A total of 45 APIs are included in these two lists (23 found in USP CC). Those APIs listed in the USP CC that are suitable for development as commercially available FDA-approved finished liquid dosage forms include clonazepam, desmopressin phytonadione, pyridoxine, rifampin, and ethambutol. Of note, desmopressin, phytonadione, and pyridoxine are available in liquid injections. Those that should remain as extemporaneously CNSPs include pyrazinamide, dapsone, diltiazem, ketoconazole, metolazone, pyrimethamine, rifabutin, bethanechol, propylthiouracil, dipyridamole, chloroquine, quinidine, temozolomide, terbutaline, tetracycline, tiagabine, dolasetron, and phenoxybenzamine.

### 5.3. Potentially Approvable Products from Extemporaneously Compounded APIs Not Included in USP CC

Zinc, buprenorphine, naltrexone, and everolimus represent API CNSP formulations available from other sources that are suitable for development and approval as commercially available finished liquid dosage forms. After appropriate testing for stability, those that could be incorporated into USP CC included amitriptyline, hydroxychloroquine, thiamine, rifaximin, valsartan, venlafaxine, buspirone, phenazopyridine, dantrolene, mexiletine, nadolol, pravastatin, topotecan, tretinoin, chlorpromazine, ethacrynic acid, flucytosine, amiloride, primaquine, procarbazine, and disopyramide. As a result of validating the monographs of over 59 listed APIs, USP identified seven CNSPs that failed stability testing using the present formulation recipe, including methimazole, phenazopyridine, probenecid, and trazodone CNSP liquids [122]. While none are considered to have high mass market potential, efforts to reformulate these APIs as stable CNSPs should be undertaken.

## 6. Discussion

The current pace of the creation and production of suitable oral dosage forms for pediatrics, whether through FDCA 505A or 503A and 503B mechanisms, could in part represent what some would call an “unfulfilled’ promise involving personalized medicine [123]. Efforts to develop oral liquid formulations for vulnerable populations, including pediatric and geriatric patients, especially for rare diseases, must continue to accelerate so that the uncertainties associated with off-label drug preparation and use are minimized and therapeutic benefits are optimized [124]. Moreover, patients and their families need assurances that formulations prepared for them have scientific evidence of safety and effectiveness based on governmental approval or professional authorization mechanisms. This is especially true with the emergence of targeted therapies for childhood cancers. For example, the BCR-ABL1 target tyrosine kinase inhibitor (TKI) imatinib was the only TKI approved for front-line treatment of pediatric patients with chronic myeloid leukemia (CML) in chronic phase (CML-CP) until dasatinib demonstrated superior efficacy [125]. A bioequivalence study involving 50 mg dispersable tablets and a 10 mg/mL suspension conducted in adults demonstrated that the bioavailability of dasatinib powder for oral suspension (PFOS) was 19% lower than that of dasatinib tablets. However, while available in oral tablets strengths ranging from 20 to 140 mg, the innovator company markets neither PFOS nor dispersible tablet formulations [126]. Further, the labeling of the product prohibits tablet crushing, making an extemporaneous oral formulation “off-label” [127]. Table 6 lists common pediatric cancers treated with targeted therapies and the availability of manufactured or compounded oral liquid formulations. This list reveals the commercial and professional gaps in the development and availability of high-quality formulations for many commonly used targeted therapies from the published scientific literature and routine clinical practice.

## 7. Summary

The purpose of this work was to evaluate the suitability of recent FDA-approved and marketed oral liquid, powder, or granule products; to identify the next group of APIs with potential for mass marketing and FDA approval; and to propose CNSPs that should be developed as approved and manufactured products, as well as those that should continue to be extemporaneously prepared. A total of 25 APIs with the highest potential for mass marketing as FDA-approved products were identified. Table 7 summarizes the remaining 48 identified APIs based on their potential for further development, either as manufactured products (n = 10) and compounded preparations (n = 38). Six USP CC-listed APIs are suitable for development and approved as commercially available FDA-approved finished liquid dosage forms, and four non-USP CC-listed CNSPs should be developed as approved mass manufactured products. Those CNSPs listed in USP CC without high mass marketing and approval potential have also been identified, as well as APIs that USP should be considered for addition to the CC, perhaps through active solicitation for the formulation monograph donation program (CPMDonate@usp.org) [138]. The current state of development of formulations for targeted therapies represents an identified gap in personalized treatments for vulnerable populations (Table 6). Through this identification and categorization process, the authors encourage industry, government, and the professions to continue to work together to improve the likelihood that patients will receive high-quality standardized extemporaneously prepared CNSPs and FDA-approved mass manufactured products.

## Figures and Tables

**Table 1:** FDA-approved commercially available finished liquid dosage forms from the 2016 AA list.

Active Pharmaceutical Ingredient	Brand Name	Strength and Dosage Form	NDA#	Company and Year of Approval
lisinopril	Qbrelis™	1 mg/mL oral solution	208401	Azurity, Woburn, MA, USA—2016
spironolactone	Carospir™	5 mg/mL oral suspension	209478	CMP Pharma, Farmville, NC, USA—2017
metoprolol succinate	Kapspargo™	25, 50, 100, and 200 mg extended release capsules	210428	Sun Pharma, Mumbai, India—2018
amlodipine benzoate	Katerzia™	1.3 mg/mL oral suspension (=1 mg amlodipine)	211340	Azurity—2019
baclofen	Ozobax™	1 mg/mL oral solution	208193	Metacel, Athens, GA, USA—2019
sildenafil	Revatio™ and generics	10 mg/mL powder for oral suspension	203109	Pfizer, Brooklyn, NY, USA, Cipla USA, Warren, NJ, USA and Novadoz, Piscataway, NJ, USA—2017–2019
levothyroxine sodium	Tirosint-SOL™	13, 25, 50, 75, 88, 100, 112, 125, 137, 150, 175, and 200 mcg/mL unit dose oral solution	206977	IBSA Pharma, Parsippany, NJ, USA—2019
amlodipine besylate	Norliqva™	1.385 mg/mL oral solution (=1 mg/mL amodipine)	214439	CMP Pharma—2022

**Table 2:** Suitability of recently approved and marketed manufactured liquids for children.

Product	Excipients Listed in the Package Insert	Rationale for Caution
Kapspargo™ (metoprolol succinate) sprinkles 25, 50, 100, 200 mg capsules	ethyl cellulose; hypromellose (hydroxypropyl methylcellulose); polyethylene glycol 400; polyethylene glycol 6000; sugar spheres (corn starch and sucrose); talc; triethyl citrate	Fixed dose may not be suitable for neonates
Qbrelis™ (lisinopril) 1 mg/mL oral liquid	water; xylitol; sodium citrate; citric acid; sodium benzoate; hydrochloric acid; sodium hydroxide	Contains sodium benzoate
Katerzia™ (amlodipine benzoate) 1 mg/mL oral suspension	citric acid monohydrate; silicone dioxide; hypromellose; maltodextrin; polysorbate 80; sodium benzoate; sodium citrate; sodium hydroxide; sucralose; water	Contains sodium benzoate and sucralose
Carospir™ (spironolactone) 5 mg/mL oral suspension	xanthan gum; dimethicone; sorbic acid; potassium sorbate; saccharin sodium; anhydrous citric acid; trisodium citrate dihydrate; ammonium glycyrrhizate (licorice); glycerin; water	Contains ammonium glycyrrhizate and saccharin
Ozobax™ (baclofen) 1 mg/mL oral solution	anhydrous citric acid; glycerin; methylparaben; propylparaben; trisodium citrate dihydrate; sucralose; water	Contains methyl- and propylparaben and sucralose
Revatio™ (sildenafil citrate) 10 mg/mL powder for oral liquid suspension	micronized cellulose; anhydrous dibasic calcium phosphate; croscarmellose sodium; magnesium stearate; hypromellose; titanium dioxide; lactose monohydrate; triacetin	Contains lactose; not labeled for children
Tirosint-SOL™—unit dose oral solution (levothyroxine sodium)—12 strengths between 13 and 200 mcg/mL	glycerin; water	Many endocrinologists prefer crushing and dissolving tablets; multiple strengths may lead to medication errors

**Table 3:** FDA-approved and marketed in finished oral liquid or granule dosage forms—2014 to present.

Active Pharmaceutical Ingredient	Brand Name	NDA#	US-FDA Approval Year	Labeled Pediatric Indication (<12 Years of Age)
glycopyrrolate	Cuvposa^™^	022571	2018	Yes
cannabidiol	Epidiolex^™^	210365	2018	Yes
vancomycin	Firvanq^™^	209910	2018	Yes
mercaptopurine	Purixan^™^	205919	2014	Yes
aprepitant	Emend^™^	207865	2015	Yes
rivaroxaban	Xarelto^™^	202439	2021	Yes
methotrexate	Xatmep^™^	208400	2017	Yes
hydrocortisone	Alkindi^™^	213876	2020	Yes
deflazacort	Emflaza^™^	208685	2017	Yes
dronabinol	Syndros^™^	205525	2016	Yes
fenfluramine	Fintepla^™^	212102	2020	Yes
tacrolimus	Prograf^™^	210115	2019	Yes
tofacitinib	Xeljanz^™^	213082	2020	Yes
**tramadol**	**Qdolo^™^**	**214044**	**2020**	**No**
stiripentol	Diacomit^™^	207223	2018	Yes
**colchicine**	**Gloperba^™^**	**210942**	**2019**	**No**
**celecoxib**	**Elyxyb^™^**	**212157**	**2020**	**No**
**sodium zirconium cyclosilicate**	**Lokelma^™^**	**207078**	**2018**	**No**
triheptanoin	Dojolvi^™^	213687	2020	Yes
topiramate	Eprontia^™^	214679	2021	Yes

**Table 5:** APIs with the highest potential for development as commercially available FDA-approved finished liquid dosage forms by BCS class.

Active Pharmaceutical Ingredient	Biopharmaceutical Classification (BCS)	Potential Pediatric Indication
clonidine	III	ADHD, neonatal abstinence syndrome, hypertension
hydroxyurea	I	Sickle cell anemia
hydrochlorothiazide	IV	Hypertension, nephrolithiasis, diabetes insipidus
amiodarone	II	Supraventricular tachycardia (SVT), atrial flutter
carvedilol	II	Heart failure, dilated cardiomyopathy
hydralazine	III	Hypertension
isradipine	II	Hypertension
allopurinol	I	Tumor lysis syndrome, urate nephropathy, hyperuricemia
flecainide	I	SVT, atrial fibrillation
quetiapine	II	Psychosis, delirium
apixaban	III	Venous thromboembolism
atenolol	III	Hypertension
cyclophosphamide	I	Sarcoma, brain tumor, vasculitis, systemic lupus erythematosus, nephrotic syndrome, juvenile rheumatoid arthritis
losartan	II	Hypertension
ganciclovir	III	Cytomegalovirus prevention and treatment
azathioprine	IV	Solid organ transplantation, inflammatory bowel disease, juvenile idiopathic arthritis, lupus nephritis, autoimmune hepatitis

**Table 6:** Selected pediatric cancers treated with targeted therapies and availability of oral liquids.

Active Pharmaceutical Ingredient	Concentration (mg/mL) Studied	Indication	Oral Liquid or Powder for Reconstitution Marketed (M) or Compounded (C)?
dasatinib [127]	10	Chronic myeloid leukemia (CML)	No
imatinib mesylate [128]	30	CML	C
nilotinib [129]	n.a.	CML	No
midostaurin [130]	25	Acute myeloid leukemia (AML)	No
vorinostat [131]	50	Cutaneous T-cell lymphoma	C
larotrectinib [132]	20	Tropomyosin receptor kinase-positive solid tumors	M
crizotinib [133]	25	ALK+ anaplastic large cell lymphoma (ALCL)	No
pazopanib [134]	50	Solid tumors in children (sarcoma, neuroblastoma, Wilms, osteosarcoma, and brain)	No
selumetinib [135]	granule	Neurofibromatosis type 1	No (in development)
trametinib [136]	0.05	Unresectable or metastatic melanoma	No
dabrafenib [137]	n.a.	Unresectable or metastatic melanoma	No

Note: n.a. = not available.

**Table 7:** APIs with potential for development as manufactured products or compounded preparations.

USP CC-Listed APIs		Non-USP CC-Listed APIs	
with Mass Market Potential	without Mass Market Potential	with Mass Market Potential	without Mass Market Potential
desmopressin	pyrazinamide	zinc	amitriptyline
phytonadione	dapsone	buprenorphine	hydroxychloroquine
pyridoxine	diltiazem	naltrexone	thiamine
rifampin	ketoconazole	everolimus	rifaxamin
ethambutol	metolazone		valsartan
clonazepam	pyrimethamine		venlafaxine
	rifabutin		buspirone
	bethanechol		dantrolene
	propylthiouracil		mexiletine
	dipyridamole		nadolol
	chloroquine		pravastatin
	quinidine		topotecan
	temozolomide		tretinoin
	terbutaline		chlorpromazine
	tetracycline		ethacrynic acid
	tiagabine		flucytosine
	dolasetron		amiloride
	phenoxybenzamine		primaquine
			procarbazine
			disopyramide

USP—United States Pharmacopeia; CC—compounding compendium; API—active pharmaceutical ingredient.

## Data Availability

Not applicable.
